# Acrylamide-Induced Changes in the Pituitary Adenylate Cyclase-Activating Polypeptide (PACAP) Immunoreactivity in Small Intestinal Intramural Neurons in Pigs

**DOI:** 10.3390/ijerph20043272

**Published:** 2023-02-13

**Authors:** Aleksandra Karpiesiuk, Jarosław Całka, Katarzyna Palus

**Affiliations:** Department of Clinical Physiology, Faculty of Veterinary Medicine, University of Warmia and Mazury in Olsztyn, Oczapowskiego Str. 13, 10-718 Olsztyn, Poland

**Keywords:** acrylamide, PACAP, enteric neurons, small intestine, pig

## Abstract

Background: A particularly pressing problem is determining consumer-safe doses of potentially health- and life-threatening substances, such as acrylamide. The aim of the study was to determine how acrylamide affects the pituitary adenylate cyclase-activating polypeptide (PACAP)-immunoreactive intramural neurons in the small intestine of sexually immature gilts. Methods: The study was conducted on 15 sexually immature Danish gilts receiving for 28 days empty gelatin capsules or acrylamide in low (0.5 µg/kg of body weight (b.w.)/day) and high (5 µg/kg b.w./day) doses. After euthanasia, intestinal sections were stained using the double immunofluorescence staining procedure. Results: Studies have shown that oral administration of acrylamide in both doses induced a response of intramural neurons expressed as an increase in the population of PACAP-immunoreactive neurons in the small intestine. In the duodenum, only in the myenteric plexus (MP) was an increase in the number of PACAP-immunoreactive (IR) neurons observed in both experimental groups, while in the outer submucous plexus (OSP) and inner submucous plexus (ISP), an increase was noted only in the high-dose group. In the jejunum, both doses of acrylamide led to an increase in the population of PACAP-IR neurons in each enteric plexus (MP, OSP, ISP), while in the ileum, only supplementation with the higher dose of acrylamide increased the number of PACAP-IR enteric neurons in the MP, OSP, and ISP. Conclusions: The obtained results suggest the participation of PACAP in acrylamide-induced plasticity of enteric neurons, which may be an important line of defence from the harmful action of acrylamide on the small intestines.

## 1. Introduction

Ensuring food safety has become a high priority for developed societies. Despite the numerous legal regulations in this regard, there are still many issues of concern. A particularly pressing problem is determining consumer-safe doses of potentially health- and life-threatening substances. Research conducted to date clearly demonstrates that acrylamide (ACM) is a toxic substance with negative effects on human and animal health. ACM has been classified into group 2A as a substance that is probably carcinogenic and mutagenic to humans by the International Agency for Research on Cancer (IARC) [1]. Moreover, it exhibits neurotoxic effects, e.g., it affects the energy metabolism of the nerves, disturbs the ionic balance, and inhibits axonal transport through binding with microtubules. ACM has also been demonstrated to induce oxidative stress and disturb synaptic vesicle synthesis, which results in ultrastructural damage (the development of profound dysfunction of the cell membrane) and functional changes (e.g., reduced neurotransmitter release) [2,3,4].

The World Health Organization (WHO) has determined that the daily intake of ACM by an adult ranges from 0.3 to 0.8 µg/kg body weight (b.w.)/day [5], and it is even greater in children. Children are particularly vulnerable to the adverse effects of ACM due to their low body weight and high consumption of products containing ACM [5]. It was also demonstrated that the toxic effect of ACM increases with an increase in both the dose and exposure time. In the current study, low ACM doses were used, i.e., 0.5 µg/kg b.w./day, corresponding to the total daily intake (TDI), and 10-fold higher doses for a period of 4 weeks, regarded as chronic exposure of pigs to this substance.

The gastrointestinal tract serves important functions in the functioning of the body, e.g., food intake, food digestion, nutrient absorption, and excretion. Moreover, it provides the first protective barrier against toxic substances contained in food. An important role in fulfilling this protective function is played by the intramural plexuses of the enteric nervous system (ENS). The ENS is composed of clusters of nerve cells forming intestinal plexuses, nerve connections between the intestinal ganglia, and nerve fibers supplying effectors, inter alia muscles of the intestinal wall, intestine-lining epithelium, internal blood vessels, and endocrine cells [6,7,8]. The ENS plays an essential role in carrying out physiological processes in the gastrointestinal tract, e.g., determining gastrointestinal movement patterns, controlling gastric acid secretion, regulating fluid flow through the lining epithelium, altering local blood flow, and interacting with the immune and endocrine systems of the gut [6].

One of the neuroactive substances synthesized in enteric neurons is pituitary adenylate cyclase-activating polypeptide (PACAP). It plays an important role in the regulation of physiological functions of the gastrointestinal tract: it stimulates the secretion of digestive juices and the release of hormones and regulates smooth muscle contraction, local blood flow, and cell migration and proliferation [9,10,11,12]. Moreover, many reports have confirmed the involvement of PACAP in pathological processes within the gastrointestinal tract, e.g., in inflammation, neuronal damage, diabetes, poisoning, and neuroplastic processes [11,12,13,14,15,16,17].

Previous studies on the effect of acrylamide on the gastrointestinal tract showed that acrylamide changed the abundance of gut flora in mice [18], led to morphological changes in the small intestine of guinea pigs [19], mice [20], rats [20], and pigs [21], and increased the levels of nitric oxide, DNA damage markers, pro-inflammatory cytokines, and oxidative stress parameters in the rats’ intestines [22]. The aim of the study was to determine how ACM affects the PACAP-immunoreactive (IR) intramural neurons in the small intestine of sexually immature gilts. Since pigs are embryologically, anatomically, and physiologically similar to humans, their significance in all kinds of biomedical research (including studies on the gastrointestinal tract) is enormous [23]. The most important similarities between the pig and human digestive tracts are: the structure of the villi and the types of cells that constitute the intestinal epithelium, the ratio of intestinal length per kilogram of body weight, the comparable transit time, and analogous digestive and absorptive processes [24]. Pigs are omnivores and have similar neonatal gut development and intestinal immune processes [23]. It is also worth emphasizing that the porcine ENS is anatomically and functionally similar to the human ENS, in contrast to rodents [8]. Thus, the results obtained may provide a starting point for further clinical research.

## 2. Materials and Methods

The study was carried out after obtaining approval from the Local Animal Research Ethics Committee in Olsztyn (approval No 11/2017). The study was conducted on 15 sexually immature Danish gilts (8 weeks, approx. 20 kg b.w.). During the experiment, the gilts were kept in age-appropriate animal pens and fed commercial pig feed. They had constant access to water. Following the acclimatization period, the animals were divided into three groups, with five heads in each. Control group (C) received empty gelatin capsules. Experimental group 1 (E1) received a low acrylamide dose of 0.5 µg/kg b.w./day, corresponding to the total daily intake (TDI) (>99%; Sigma-Aldrich, Saint Louis, MO, USA). Experimental group 2 (E2) received a high acrylamide dose of 5 µg/kg b.w./day. The animals were weighed weekly in order to determine the ACM dose for supplementation. The capsules were administered during the morning feeding for 28 days. After this time, all the pigs were euthanized. Then, a midline laparotomy was performed and all sections of the gastrointestinal tract were removed. After that, the duodenum (located 10 cm caudal to the gastric pylorus), jejunum (located about 40 cm after the gastric pylorus), and ileum (located 2 cm before the ileocecal valve) tissues were collected for further research. The tissues were immersion-fixed by the immersion in 4% paraformaldehyde (pH 7.4) for 1 h at room temperature and then rinsed three times in 0.1 M phosphate buffer (pH 7.4) for 72 h, with the buffer changed on a daily basis. After these steps, the tissues were transferred to an 18% buffered sucrose solution (pH 7.4) for two weeks. In the next step, the tissues were frozen at a temperature of −30 °C. Subsequently, the intestines were cut into serial sections (with a thickness of 14 µm) using a cryostat.

Intestinal sections prepared in this way were stained using the double immunofluorescence staining procedure (as previously described by Palus et al. [25]). On the first day of staining, the sections were dried for 45 min at room temperature. They were then rinsed three times for 10 min in 0.1 M buffer solution (PBS, pH 7.4), and the tissues were subsequently blocked in a blocking mixture (10% normal goat serum Sigma, St. Louis, MO, USA, Cat. Nr. G9023, in PBS with 0.3% Triton X-100 Sigma, St. Louis, MO, USA, and 1% bovine serum albumin (BSA); Sigma, St. Louis, MO, USA). After blocking, the specimens were re-rinsed in PBS (3 × 10 min). A primary antibody mixture comprising Hu C/D was applied (mouse polyclonal, Invitrogen, Waltham, MA, USA, Cat. No A-21271, lot nr. 1,900,217 working dilution: 1:1000, used as a pan-neuronal marker), and the pituitary adenylate cyclase-activating peptide (PACAP, guinea pig polyclonal, Peninsula, San Carlos, CA, USA, Cat. No T-5039, lot nr. 040119-1 working dilution: 1:2000) was incubated overnight at room temperature in a humid chamber. On the second day, the specimens were rinsed in PBS (3 × 10 min), and then secondary antibodies were applied (Alexa Fluor 488 nm donkey anti-mouse, ThermoFisher Scientific, Waltham, MA, USA; Cat. No A21202; lot nr. 1,696,430 working dilution: 1:1000, and Alexa Fluor 546 nm donkey anti-guinea pig, ThermoFisher Scientific, Waltham, MA, USA, Cat. No A11074; lot nr. 1,073,002 working dilution: 1:1000), incubated for 1 h. After this time, the specimens were re-rinsed in PBS (as above). After the completion of staining, the sections were covered with a mixture of glycerol and carbonate buffer (pH 8.4) and covered with a cover glass. The verification of specificity included the pre-absorption, substitution, and omission tests, with all the above tests yielding a negative result. The neurons immunoreactive towards the active substances under study were counted in all sections of the small intestine, i.e., the duodenum, jejunum, and ileum. They were analyzed using an Olympus BX51 epifluorescence microscope. For each type of intestinal plexus (myenteric plexus (MP), outer submucosal plexus (OSP), and inner submucosal plexus (ISP)), at least 500 neurons immunoreactive towards Hu C/D (pan-neuronal marker) were counted, while the number of PACAP-positive intestinal neurons was referred to as a percentage of Hu C/D-positive neurons. The analyzed sections were located at least 200 µm apart. The results obtained were subjected to statistical analysis with the Statistica 13 program (Stat Soft Inc., Tulsa, OK, USA) using a one-way analysis of variance (ANOVA) with Dunnett’s test and expressed as a mean ± standard error of the mean (SEM) (* *p* < 0.05, ** *p* < 0.01, *** *p* < 0.001).

## 3. Results

This study demonstrated the presence of PACAP-immunoreactive neurons in all types of intramural plexuses (MP, OSP, ISP) in the small intestine region of pigs in the physiological condition and following supplementation with a low and high acrylamide dose.

### 3.1. The Duodenum

**Figure 1:**
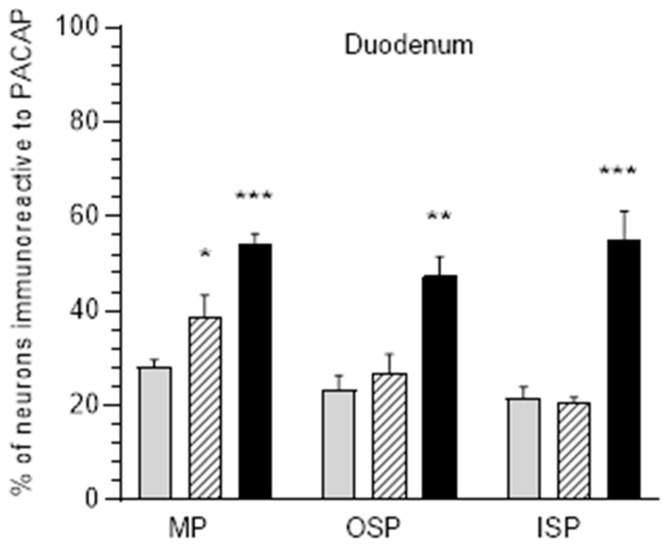
Diagrams illustrate percentage changes of PACAP-positive neuronal cells in the myenteric plexus (MP), outer submucosal plexus (OSP), and inner submucosal plexus (ISP), in the duodenum in pigs. Grey bars indicate the control group, hatched bars E1 group, black bars E2 group. * *p* < 0.05, ** *p* < 0.01, *** *p* < 0.001 indicate changes in the expression of PACAP in experimental groups with respect to control group.

**Figure 2:**
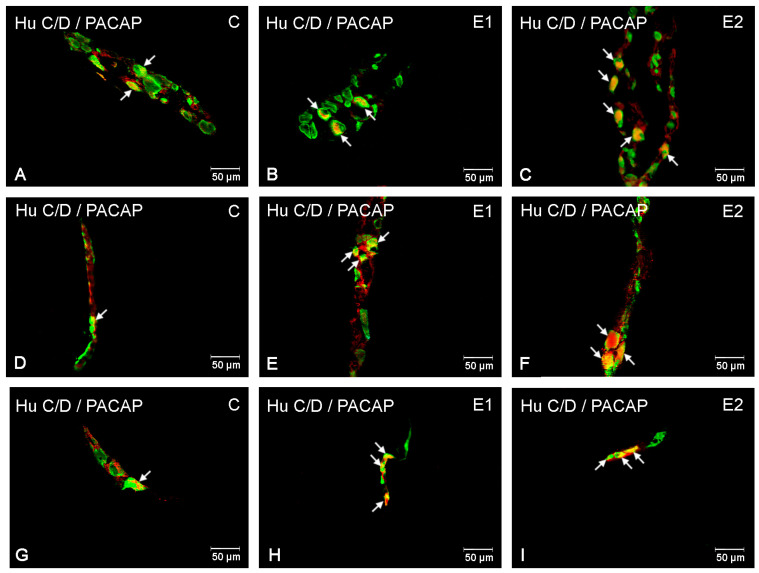
Microscopy pictures presenting the distribution of PACAP-immunoreactive neuronal cell bodies (red) in the duodenum in control animals (C) and animals from experimental group 1 (E1) and 2 (E2). Hu C/D was used as a pan-neuronal marker (green). All pictures are the result of a computer overlapping of two channels (red and green). Pictures (**A**–**C**) illustrate neurons in the myenteric plexus; pictures (**D**–**F**) illustrate neurons in the outer submucosal plexus; pictures (**G**–**I**) illustrate neurons in the inner submucosal plexus. Enteric neurons immunoreactive to Hu C/D and PACAP are indicated with arrows.

In the physiological condition, PACAP-positive neurons were most abundant in the myenteric plexuses, where they accounted for 28.27 ± 1.45% of Hu C/D-immunoreactive neurons. A slightly less abundant population of PACAP-IR neurons was revealed in submucosal plexuses: 23.4 ± 3.03% in OSP and 21.59 ± 2.42% in ISP (Figure 1 and Figure 2A,D,G). The administration of capsules containing acrylamide resulted in a change in the number of PACAP-IR neurons within the duodenum. A low acrylamide dose resulted in an increase in the PACAP-positive neuron population only in the MP (up to 38.91 ± 4.5%) (Figure 2B). However, a high acrylamide dose increased the PACAP-IR neuron population in both the MP (up to 54.37 ± 1.97%) (Figure 2C) and the submucosal plexuses: in OSP (up to 47.42 ± 4.19%) (Figure 2F) and in ISP (up to 54.88 ± 6.24%) (Figure 2I).

### 3.2. The Jejunum

**Figure 3:**
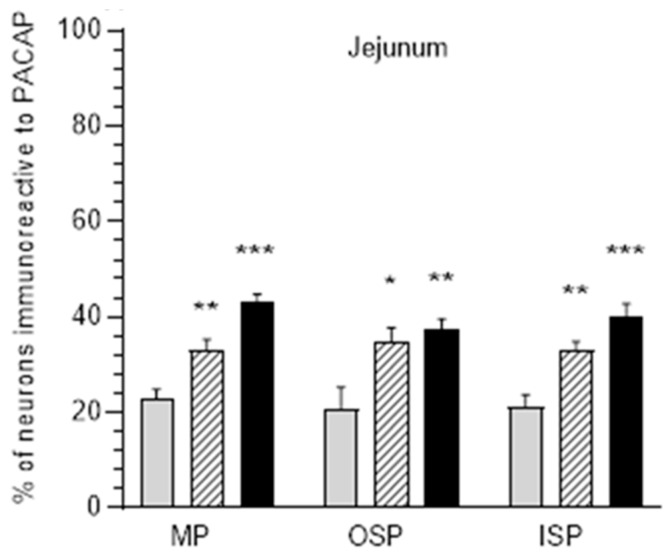
Diagrams illustrate percentage changes of PACAP-positive neuronal cells in the myenteric plexus (MP), outer submucosal plexus (OSP), and inner submucosal plexus (ISP) in the jejunum in pigs. Grey bars indicate the control group, hatched bars E1 group, black bars E2 group. * *p* < 0.05, ** *p* < 0.01, *** *p* < 0.001 indicate changes in the expression of PACAP in experimental groups with respect to control group.

**Figure 4:**
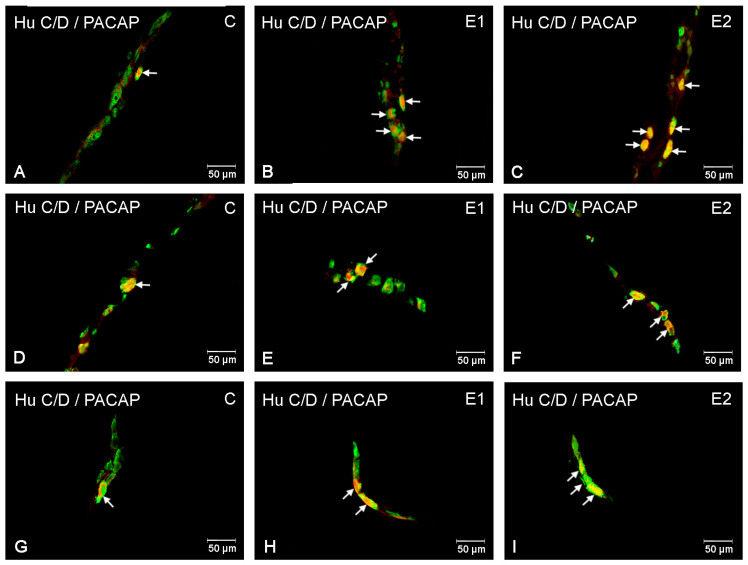
Microscopy pictures presenting the distribution of PACAP-immunoreactive neuronal cell bodies (red) in the jejunum in control animals (C) and animals from experimental group 1 (E1) and 2 (E2). Hu C/D was used as a pan-neuronal marker (green). All pictures are the result of a computer overlapping of two channels (red and green). Pictures (**A**–**C**) illustrate neurons in the myenteric plexus; pictures (**D**–**F**) illustrate neurons in the outer submucosal plexus; pictures (**G**–**I**) illustrate neurons in the inner submucosal plexus. Enteric neurons immunoreactive to Hu C/D and PACAP are indicated with arrows.

As in the duodenum, in the control group, the largest population of PACAP-IR neurons was found in MP (22.68 ± 2.18%). In the submucosal plexuses, these values were similar and accounted for 20.70 ± 4.65% in OSP and 21.7 ± 2.59% in ISP, respectively (Figure 3 and Figure 4A,D,G). Both of the acrylamide doses applied significantly increased the number of PACAP-positive neurons in all types of intramural ganglia subjected to analysis. In MP, a lower acrylamide dose increased the PACAP-IR neuron population to 33.14 ± 2.19% (Figure 4B), while a higher dose increased it to 42.32 ± 1.40% (Figure 4C); in OSP, it was increased to 34.82 ± 2.95% for a low dose (Figure 4E) and to 37.38 ± 2.24% for a high dose (Figure 4F); in ISP, it was increased to 33.1 ± 1.79% (Figure 4H) and to 39.9 ± 2.91% (Figure 4I), respectively.

### 3.3. The Ileum

**Figure 5:**
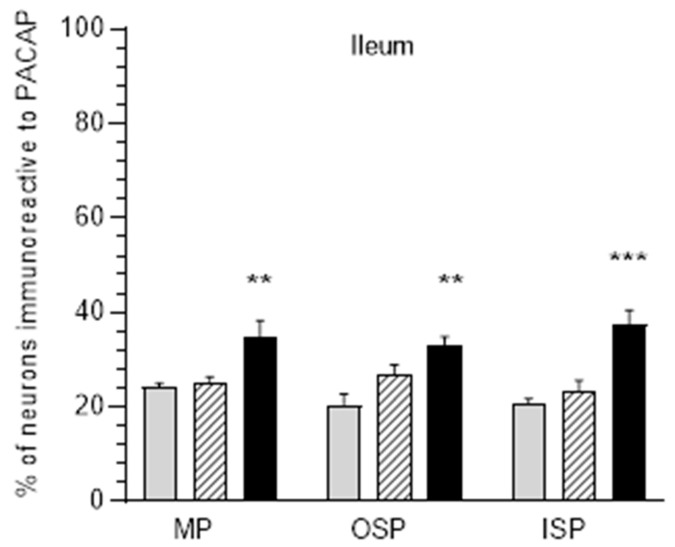
Diagrams illustrate percentage changes of PACAP-positive neuronal cells in the myenteric plexus (MP), outer submucosal plexus (OSP), and inner submucosal plexus (ISP) in the ileum in pigs. Grey bars indicate the control group, hatched bars E1 group, black bars E2 group, ** *p* < 0.01, *** *p* < 0.001 indicate changes in the expression of PACAP in experimental groups with respect to control group.

**Figure 6:**
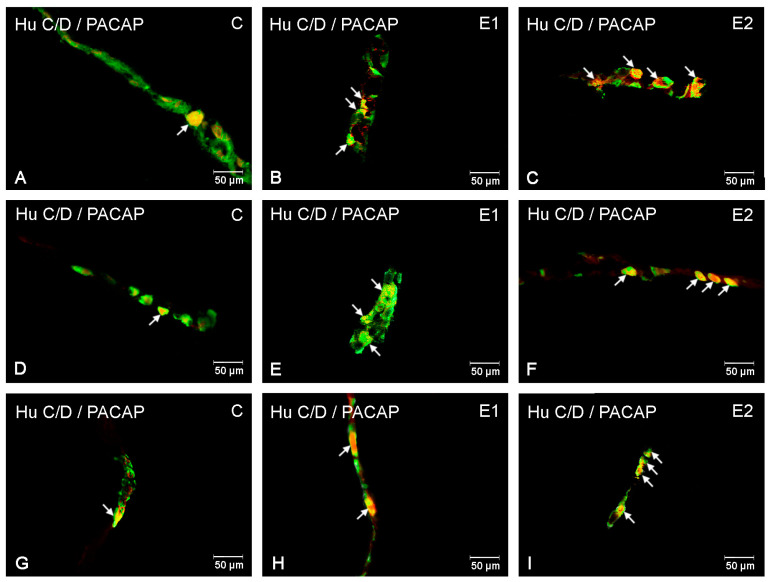
Microscopy pictures presenting the distribution of PACAP-immunoreactive neuronal cell bodies (red) in the ileum in control animals (C) and animals from experimental group 1 (E1) and 2 (E2). Hu C/D was used as a pan-neuronal marker (green). All pictures are the result of a computer overlapping of two channels (red and green). Pictures (**A**–**C**) illustrate neurons in the myenteric plexus; pictures (**D**–**F**) illustrate neurons in the outer submucosal plexus; pictures (**G**–**I**) illustrate neurons in the inner submucosal plexus. Enteric neurons immunoreactive to Hu C/D and PACAP are indicated with arrows.

In control animals, in the ileum region, PACAP was most abundant in myenteric plexuses (24.2 ± 0.82%), while a slightly smaller population of PACAP-IR neurons was noted in ISP (20.9 ± 0.9%) and OSP (20.49 ± 2.27%) (Figure 5 and Figure 6A,D,G). The acrylamide supplementation increased the number of PACAP-positive neurons in all three plexuses (MP, OSP, ISP) only in the group receiving high doses. In MP, an increase to 34.76 ± 3.48% was noted (Figure 6C), while in OSP the increase was 33.18 ± 1.68% (Figure 6F), and in ISP the increase was 37.59 ± 2.85% (Figure 6I).

## 4. Discussion

The study confirmed the presence of PACAP-immunoreactive neurons in the intramural plexuses of the ENS in the small intestine in the pig, which is consistent with previous reports, as the presence of PACAP was confirmed in the ENS of other animal species: rats [10,26,27], mice [28], cats [28], ferrets [28], guinea pigs [29,30], sheep [28,31], and humans [30]. The research conducted so far has demonstrated that PACAP serves numerous functions in the gastrointestinal tract, particularly in the small intestine. Its most important function is to regulate intestinal motility, i.e., the stimulation and/or inhibition of spontaneous intestinal contractility. Another physiological function of PACAP is the stimulation of electrogenic ion secretion in the jejunum of rats and humans [32,33] and bicarbonate secretion in the duodenum of rats [34], as well as cholecystokinin secretion in the small intestine of rats and mice [35,36,37]. Moreover, it exhibits properties which inhibit the secretion of 5-hydroxytryptamine in the small intestine of mice and rats [35,36,37]. It is commonly known that the myenteric ganglion is responsible for the regulation of the intestinal motor function, while the submucosal ganglia regulate secretory processes. Having considered the above physiological functions of PACAP and its presence in all types of intestinal plexuses, it can be concluded that PACAP is an important regulator of physiological functions within the small intestine of the pig.

Supplementation of gilts with acrylamide brought about significant changes in the population of PACAP-positive neurons in the small intestine. Previous research into ENS plasticity has shown that disturbance of homeostasis resulting from inflammation, neuronal damage, or poisoning within the gastrointestinal tract can induce a neuronal response expressed as changes in excitability, structural change, and a change in the neurochemical phenotype of nerve cells [38,39,40], expressed as a reduction or an increase in the synthesis of neuroactive substances in enteric neurons. Previous research also showed that PACAP is one of the most important neuropeptides with protective effects [14,15,16,17,26]. The increase in the secretion of this neuropeptide following ACM supplementation suggests that it can play an important role in pathological processes occurring within the gastrointestinal tract, which is confirmed by previous research. Changes in the PACAP-positive neuron population in the ENS plexuses in the duodenum and jejunum regions of the pig have been described as an effect of the use of non-steroidal anti-inflammatory drugs (NSAIDs). Following naproxen, indomethacin, and aspirin supplementation, an increase in the number of PACAP-IR neurons in all three types of ENS plexuses (MP, OSP, ISP) was observed [22,23,24,25,26,27,28,29,30,31,32,33,34,35,36,37,38,39,40,41,42,43]. Similarly, an increase in the number of PACAP-positive neurons in the submucosal ganglion was observed in the course of gastric hyperacidity induced by hydrochloric acid infusion [44]. Furthermore, Gonkowski and Całka [11] demonstrated an increase in the number of PACAP-IR neurons in the pathological conditions of the porcine descending colon, i.e., chemical inflammation (CI), proliferative enteropathy (PE), and axotomy (AA). They observed an increase in the PACAP-positive neuron population in myenteric ganglia in all experimental groups, yet the greatest changes were observed during PE. In OSP, coding changes were observed in CI and PE, while in ISP, changes occurred in all the conditions under study [11]. In turn, diabetes induced changes in the population of PACAP-positive neurons located in different sections of the porcine gastrointestinal tract. In the stomach, a statistically significant increase in the number of neurons showing immunoreactivity towards PACAP has been demonstrated in the submucosal plexus. In the duodenum, an increase has been demonstrated in ISP and MP, in the jejunum in OSP and MP, and in the ileum in each of the intramural plexuses under study (ISP, OSP, and MP). Similarly, in the descending colon, a significant increase was noted in each of the plexuses under study (ISP, OSP, MP) [13]. In turn, the administration of a low dose of lipopolysaccharides (LPSs) to *Salmonella Enteritidis* resulted in a drop in the PACAP-positive neuron population in the OSP and ISP neural plexuses within the duodenum region of the pig [45]. Following the administration of capsaicin to rats, a drop in the number of PACAP-IR neurons was observed in the esophagus, cardia, fundus, and corpus of the stomach and in the colon [26]. However, research into carcinoma of the human large intestine has demonstrated no changes in the PACAP-positive neuron population [16]. This suggests that the role of PACAP in the gastrointestinal tract is multi-directional and determined by the pathological condition and the section of the gastrointestinal tract subjected to analysis. In the current study, an increase in the PACAP-IR neuron population was also noted in each of the intestinal plexuses under study (MP, OSP, ISP) in the small intestinal region of the pig as a result of ACM supplementation. Although further efforts are needed to fully recognize the mechanism(s) of the observed changes, the increase in the number of PACAP-IR neurons over the course of acrylamide intoxication may be the result of an increased synthesis of this neuropeptide at various stages of this process (transcription, translation, or changes in the activity of enzymes involved in the synthesis) as well as changes at the axonal transport stage (neurotransmission disorders).

It is also worth noting that PACAP serves an important role in the protection of neurons against oxidative stress [46]. An in vivo study on zebrafish demonstrated that PACAP protects the hair cells against oxidative stress by attenuating apoptosis [47]. Additionally, it reduces the adverse effect of oxidative stress on the intestinal epithelial cells having high turnover (INT 407) [48]. Previous research demonstrated that ACM induces oxidative stress in the bodies of animals and humans. Ibrahim and Ibrahem [49] demonstrated that exposure to ACM increases the oxidative stress marker levels in a catfish species (Clarias gariepinus). Patients who received an ACM-rich diet exhibited increased production of reactive oxygen radicals by leukocytes [50]. Therefore, one of the reasons for the increase in the PACAP-positive neuron population, noted in the current study, could be a response of ENS neurons to acrylamide-induced oxidative stress. However, in order to confirm this hypothesis, further detailed research is needed.

PACAP also plays an important role in the regulation of inflammatory processes in the gastrointestinal tract. The anti-inflammatory effects of PACAP have been observed in the course of acute ileitis in mice [51], and it has been demonstrated that in the course of experimentally induced subacute ileitis in mice, PACAP reduced T-lymphocyte levels and had a cytoprotective effect on the ileal epithelium, resulting in reduced synthesis of pro-inflammatory cytokines in the intestinal wall [52]. However, research into PACAP deficiency in mice has shown an altered composition of the intestinal microbiota and a complete absence of bifidobacteria, which may consequently lead to an increased frequency of intestinal disorders [53]. In contrast, the intravenous administration of PACAP-27 increased the secretion of bicarbonates which reduce inflammatory changes in the duodenum [54]. ACM is commonly known to induce inflammation, which has been observed, e.g., in research into albinotic mice receiving ACM. ACM supplementation triggered an increase in the secretion of pro-inflammatory cytokines such as tumor necrosis factor-α (TNF-α), interleukin 1 β (IL-1β), and the inducible nitric oxide synthase (iNOS) in the CNS [55]. A previous study by the authors showed [21] that exposure to ACM results in increased synthesis of pro-inflammatory cytokines (IL-1β, interleukin 6 (IL-6), TNF-α) by the ileum Peyer patches in pigs following the ACM supplementation. Moreover, a clinical study has demonstrated that high ACM doses result in an increase in the secretion of inflammatory mediators (IL-6, C-reactive protein (CRP)) and the oxidized form of cholesterol (LDH) in the human body [50]. One of the probable reasons for the changes in the number of PACAP-IR neurons observed in the present study may be the response of ENS neurons to local inflammation induced by acrylamide.

## 5. Conclusions

In summary, enteric neurons exhibit a high degree of adaptation to perturbations of homeostasis induced by agents of a potentially neurotoxic nature. The current study demonstrated, for the first time, that acrylamide brings about changes in the PACAP-IR neuron population in the small intestine of the pig. The increase in immunoreactivity towards PACAP in all types of intramural plexuses proves that legal doses of acrylamide in food affect the neurochemical properties of intestinal neurons and thus are not indifferent to the organism. Observed changes may be the result of an increased synthesis of PACAP at various stages of this process (transcription, translation, or changes in the activity of enzymes involved in the synthesis) as well as changes at the axonal transport stage (neurotransmission disorders). PACAP is probably engaged in acrylamide-induced plasticity of enteric neurons, which may be an important line of defense from the harmful action of acrylamide on small intestines. However, the issue of the exact mechanism of the harmful effects of ACM on the intestine requires further detailed research.

## Data Availability

The data presented in this study are available on request from the corresponding author.

## References

[1] Chico Galdo V., Massart C., Jin L., Vanvooren V., Caillet-Fauquet P., Andry G., Dequanter D., Friedman M., van Sande J. (2006). Acrylamide an in vitro thyroid carcinogenic agent, induces DNA damage in rat thyroid cell lines and primary cultures. Mol. Cell. Endocrinol..

[2] Kopanska M., Muchacka R., Czech J., Batoryna M., Formicki G. (2018). Acrylamide toxicity and cholinergic nervous system. J. Physiol. Pharmacol..

[3] Yousef M.I., El-Demerdash F.M. (2006). Acrylamide-induced oxidative stress and biochemical perturbations in rats. Toxicology.

[4] LoPachin R.M. (2004). The changing view of acrylamide neurotoxicity. Neurotoxicology.

[5] WHO (2002). Health Implications of Acrylamide in Food.

[6] Furness J.B. (2012). The enteric nervous system and neurogastroenterology. Review. Nat. Rev. Gastroenterol. Hepatol..

[7] Furness J.B. (2000). Types of neurons in the enteric nervous system. J. Auton. Nerv. Syst..

[8] Brown D.R., Timmermans J.P. (2004). Lessons from the porcine enteric nervous system. Neurogastroenterol. Motil..

[9] Reglodi D., Illes A., Opper B., Schafer E., Tamas A., Horvath G. (2018). Presence and Effects of Pituitary Adenylate Cyclase Activating Polypeptide Under Physiological and Pathological Conditions in the Stomach. Front. Endocrinol..

[10] Läuff J.M., Modlin I.M., Tang L.H. (1999). Biological relevance of pituitary adenylate cyclase-activating polypeptide (PACAP) in the gastrointestinal tract. Regul. Pept..

[11] Gonkowski S., Całka J. (2012). Changes in pituitary adenylate cyclase-activating Peptide 27-like immunoreactive nervous structures in the porcine descending colon during selected pathological processes. J. Mol. Neurosci..

[12] Karpiesiuk A., Palus K. (2021). Pituitary Adenylate Cyclase-Activating Polypeptide (PACAP) in Physiological and Pathological Processes within the Gastrointestinal Tract: A Review. Int. J. Mol. Sci..

[13] Palus K., Bulc M., Całka J., Zielonka Ł., Nowicki M. (2021). Diabetes Affects the Pituitary Adenylate Cyclase-Activating Polypeptide (PACAP)-Like Immunoreactive Enteric Neurons in the Porcine Digestive Tract. Int. J. Mol. Sci..

[14] Gonkowski S., Obremski K., Całka J. (2015). The Influence of Low Doses of Zearalenone on Distribution of Selected Active Substances in Nerve Fibers Within the Circular Muscle Layer of Porcine Ileum. J. Mol. Neurosci..

[15] Thoene M., Rytel L., Dzika E., Wojtkiewicz J. (2021). Increased PACAP- and DβH-Positive Hepatic Nerve Fibers after Bisphenol A Exposure. Toxics.

[16] Godlewski J., Łakomy I.M. (2010). Changes in vasoactive intestinal peptide, pituitary adenylate cyclase-activating polypeptide and neuropeptide Y-ergic structures of the enteric nervous system in the carcinoma of the human large intestine. Folia Histochem. Cytobiol..

[17] Szanto Z., Sarszegi Z., Reglodi D., Nemeth J., Szabadfi K., Kiss P., Varga A., Banki E., Csanaky K., Gaszner B. (2012). PACAP immunoreactivity in human malignant tumor samples and cardiac diseases. J. Mol. Neurosci..

[18] Wang Z., Liu H., Liu J., Ren X., Song G., Xia X., Qin N. (2021). Dietary Acrylamide Intake Alters Gut Microbiota in Mice and Increases Its Susceptibility to Salmonella Typhimurium Infection. Foods.

[19] Tomaszewska E., Dobrowolski P., Puzio I., Prost L., Kurlak P., Sawczuk P., Badzian B., Hulas-Stasiak M., Kostro K. (2014). Acrylamide-induced prenatal programming of intestine structure in guinea pig. J. Physiol. Pharmacol..

[20] Su D., Lei A., Nie C., Chen Y. (2023). The protective effect of Ganoderma atrum polysaccharide on intestinal barrier function damage induced by acrylamide in mice through TLR4/MyD88/NF-κB based on the iTRAQ analysis. Food Chem. Toxicol..

[21] Palus K., Obremski K., Bulc M., Całka J. (2019). The impact of low and high doses of acrylamide on the intramural neurons of the porcine ileum. Food Chem. Toxicol..

[22] Zhao S., Sun H., Liu Q., Shen Y., Jiang Y., Li Y., Liu T., Liu T., Xu H., Shao M. (2020). Protective effect of seabuckthorn berry juice against acrylamide-induced oxidative damage in rats. J. Food Sci..

[23] Swindle M.M., Smith A.C. (1998). Comparative anatomy and physiology of the pig. Scand. J. Lab. Anim. Sci..

[24] Gonzalez L.M., Moeser A.J., Blikslager A.T. (2015). Porcine models of digestive disease: The future of large animal translational research. Transl. Res..

[25] Palus K., Bulc M., Całka J. (2017). Changes in Somatostatin-Like Immunoreactivity in the Sympathetic Neurons Projecting to the Prepyloric Area of the Porcine Stomach Induced by Selected Pathological Conditions. Biomed. Res. Int..

[26] Hannibal J., Ekblad E., Mulder H., Sundler F., Fahrenkrug J. (1998). Pituitary adenylate cyclase activating polypeptide (PACAP) in the gastrointestinal tract of the rat: Distribution and effects of capsaicin or denervation. Cell Tissue Res..

[27] Nagahama M., Tsuzuki M., Mochizuki T., Iguchi K., Kuwahara A. (1998). Light and electron microscopic studies of pituitary adenylate cyclase-activating peptide (PACAP)—Immunoreactive neurons in the enteric nervous system of rat small and large intestine. Anat. Embryol..

[28] Sundler F., Ekblad E., Absood A., Håkanson R., Köves K., Arimura A. (1992). Pituitary adenylate cyclase activating peptide: A novel vasoactive intestinal peptide-like neuropeptide in the gut. Neuroscience.

[29] Portbury A.L., McConalogue K., Furness J.B., Young H.M. (1995). Distribution of pituitary adenylyl cyclase-activating peptide (PACAP) immunoreactivity in neurons of the guinea-pig digestive tract and their projections in the Palus^0 and colon. Cell. Tissue Res..

[30] Suzuki M., Nokihara K., Ando E., Naruse S. (1996). Immunohistochemical comparison of localization of pituitary adenylate cyclase activating polypeptide (PACAP) and vasoactive intestinal polypeptide (VIP) in the enteric nerve plexus of the guinea pig jejunum. Biomed. Pept. Proteins Nucleic Acids.

[31] Köves K., Arimura A., Vigh S., Somogyvári-Vigh A., Miller J. (1993). Immunohistochemical localization of PACAP in the ovine digestive system. Peptides.

[32] Cox H.M. (1992). Pituitary adenylate cyclase activating polypeptides, PACAP-27 and PACAP-38: Stimulators of electrogenic ion secretion in the rat small intestine. Br. J. Pharmacol..

[33] Fuchs M., Adermann K., Raab H.R., Forssmann W.G., Kuhn M. (1996). Pituitary adenylate cyclase-activating polypeptide: A potent activator of human intestinal ion transport. Ann. N. Y. Acad. Sci..

[34] Takeuchi K., Takehara K., Kato S., Yagi K. (1997). PACAPs stimulate duodenal bicarbonate secretion at PACAP receptors in the rat. Am. J. Physiol..

[35] Chang C.H., Chey W.Y., Braggins L., Coy D.H., Chang T.M. (1996). Pituitary adenylate cyclase-activating polypeptide stimulates cholecystokinin secretion in STC-1 cells. Am. J. Physiol..

[36] Racké K., Reimann A., Schwörer H., Kilbinger H. (1996). Regulation of 5-HT release from enterochromaffin cells. Behav. Brain Res..

[37] Chang C.H., Chey W.Y., Erway B., Coy D.H., Chang T.M. (1998). Modulation of secretin release by neuropeptides in secretin-producing cells. Am. J. Physiol..

[38] Ekblad E., Bauer A.J. (2004). Role of vasoactive intestinal peptide and inflammatory mediators in enteric neuronal plasticity. Neurogastroenterol. Motil..

[39] Furness J.B., Callaghan B.P., Rivera L.R., Cho H.J. (2014). The enteric nervous system and gastrointestinal innervation: Integrated local and central control. Adv. Exp. Med. Biol..

[40] Gonkowski S. (2013). Substance P as a neuronal factor in the enteric nervous system of the porcine descending colon in physiological conditions and during selected pathogenic processes. Biofactors.

[41] Czajkowska M., Całka J. (2020). Neurochemistry of Enteric Neurons Following Prolonged Indomethacin Administration in the Porcine Duodenum. Front. Pharmacol..

[42] Czajkowska M., Rychlik A., Całka J. (2020). Long-term treatment with naproxen changes the chemical coding of the porcine intramural duodenum neurons. Ann. Anat..

[43] Brzozowska M., Jana B., Całka J. (2021). Effect of NSAIDs Supplementation on the PACAP, SP- and GAL-Immunoreactive Neurons in the Porcine Jejunum. Int. J. Mol. Sci..

[44] Całka J. (2019). Increased expression of CART, Nnos, VIP, PACAP, SP and GAL in enteric neurons of the porcine stomach prepyloric region following hydrochloric acid infusion. Folia Histochem. Cytobiol..

[45] Rytel L., Wojtkiewicz J., Snarska A., Mikołajczyk A. (2020). Changes in the Neurochemical Characterization of Enteric Neurons in the Porcine Duodenum after Administration of Low-Dose Salmonella Enteritidis Lipopolysaccharides. J. Mol. Neurosci..

[46] Ohtaki H., Satoh A., Nakamachi T., Yofu S., Dohi K., Mori H., Ohara K., Miyamoto K., Hashimoto H., Shintani N. (2010). Regulation of oxidative stress by pituitary adenylate cyclase-activating polypeptide (PACAP) mediated by PACAP receptor. J. Mol. Neurosci..

[47] Kasica N., Podlasz P., Sundvik M., Tamas A., Reglodi D., Kaleczyc J. (2016). Protective Effects of Pituitary Adenylate Cyclase-Activating Polypeptide (PACAP) Against Oxidative Stress in Zebrafish Hair Cells. Neurotox. Res..

[48] Illes A., Opper B., Reglodi D., Kerenyi M., Czetany P., Boronkai A., Schafer E., Toth G., Fabian E., Horvath G. (2017). Effects of pituitary adenylate cyclase activating polypeptide on small intestinal INT 407 cells. Neuropeptides.

[49] Ibrahim M.A., Ibrahem M.D. (2020). Acrylamide-induced hematotoxicity, oxidative stress, and DNA damage in liver, kidney, and brain of catfish (Clarias gariepinus). Environ. Toxicol..

[50] Naruszewicz M., Zapolska-Downar D., Kośmider A., Nowicka G., Kozłowska-Wojciechowska M., Vikström A.S., Törnqvist M. (2009). Chronic intake of potato chips in humans increases the production of reactive oxygen radicals by leukocytes and increases plasma C-reactive protein: A pilot study. Am. J. Clin. Nutr..

[51] Heimesaat M.M., Dunay I.R., Schulze S., Fischer A., Grundmann U., Alutis M., Kühl A.A., Tamas A., Toth G., Dunay M.P. (2014). Pituitary adenylate cyclase-activating polypeptide ameliorates experimental acute ileitis and extra-intestinal sequelae. PLoS ONE.

[52] Bereswill S., Escher U., Grunau A., Kühl A.A., Dunay I.R., Tamas A., Reglodi D., Heimesaat M.M. (2019). Pituitary Adenylate Cyclase-Activating Polypeptide-A Neuropeptide as Novel Treatment Option for Subacute Ileitis in Mice Harboring a Human Gut Microbiota. Front. Immunol..

[53] Heimesaat M.M., Reifenberger G., Vicena V., Illes A., Horvath G., Tamas A., Fulop B.D., Bereswill S., Reglodi D. (2017). Intestinal Microbiota Changes in Mice Lacking Pituitary Adenylate Cyclase Activating Polypeptide (PACAP)—Bifidobacteria Make the Difference. Eur. J. Microbiol. Immunol..

[54] Yagi K., Takehara K., Kitamura M., Takeuchi K. (1998). Effects of pituitary adenylate cyclase activating polypeptide-27 on alkaline secretory and mucosal ulcerogenic responses in rat duodenum. Life Sci..

[55] Santhanasabapathy R., Vasudevan S., Anupriya K., Pabitha R., Sudhandiran G. (2015). Farnesol quells oxidative stress, reactive gliosis and inflammation during acrylamide-induced neurotoxicity: Behavioral and biochemical evidence. Neuroscience.

